# Tumor Resistance against ALK Targeted Therapy-Where It Comes From and Where It Goes

**DOI:** 10.3390/cancers10030062

**Published:** 2018-02-28

**Authors:** Geeta Geeta Sharma, Ines Mota, Luca Mologni, Enrico Patrucco, Carlo Gambacorti-Passerini, Roberto Chiarle

**Affiliations:** 1 Department of Medicine and Surgery, University of Milano-Bicocca, Monza 20900, Italy; geeta.geeta@unimib.it (G.G.S.); luca.mologni@unimib.it (L.M.); carlo.gambacorti@unimib.it (C.G.-P.); 2 Department of Molecular Biotechnology and Health Sciences, University of Turin, Turin 10124, Italy; ines.mota05@gmail.com (I.M.); enrico.patrucco@unito.it (E.P.); 3 Galkem Srl, Monza 20900, Italy; 4 Hematology and Clinical Research Unit, San Gerardo Hospital, Monza 20900, Italy; 5 Department of Pathology, Boston Children’s Hospital, Harvard Medical School, Boston, MA 02115, USA

**Keywords:** anaplastic large-cell lymphoma (ALCL), anaplastic lymphoma kinase (ALK), ALK inhibitors, non-small-cell lung cancer (NSCLC), resistance to ALK inhibitors, targeted therapies, tyrosine kinase (TK)

## Abstract

Anaplastic lymphoma kinase (ALK) is a validated molecular target in several ALK-rearranged malignancies, particularly in non-small-cell lung cancer (NSCLC), which has generated considerable interest and effort in developing ALK tyrosine kinase inhibitors (TKI). Crizotinib was the first ALK inhibitor to receive FDA approval for ALK-positive NSCLC patients treatment. However, the clinical benefit observed in targeting ALK in NSCLC is almost universally limited by the emergence of drug resistance with a median of occurrence of approximately 10 months after the initiation of therapy. Thus, to overcome crizotinib resistance, second/third-generation ALK inhibitors have been developed and received, or are close to receiving, FDA approval. However, even when treated with these new inhibitors tumors became resistant, both in vitro and in clinical settings. The elucidation of the diverse mechanisms through which resistance to ALK TKI emerges, has informed the design of novel therapeutic strategies to improve patients disease outcome. This review summarizes the currently available knowledge regarding ALK physiologic function/structure and neoplastic transforming role, as well as an update on ALK inhibitors and resistance mechanisms along with possible therapeutic strategies that may overcome the development of resistance.

## 1. Introduction

Over the last decade, the development of drugs that selectively target driver oncogenes has played an important role to establish novel treatment guidelines in the field of oncology. Unlike traditional chemo and radio-therapies that kill all rapidly dividing cells, targeted therapies are more selective and specific towards their target, exploiting the biology that drives the growth of tumor cells such as genetic deletions, chromosomal rearrangements and point mutations. Furthermore, targeted therapies have significantly impacted outcomes in terms of prolonged survival and a better quality of life for cancer patients. 

Imatinib, a small molecule tyrosine kinase inhibitor (TKI) developed to treat chronic myeloid leukemia (CML) patients bearing t(9;22)(q34;q11), was the first breakthrough in the journey of target therapies [1]. Five years follow-up studies have shown that patients treated with imatinib achieved molecular responses and overall survival not different from the general population [2]. 

Another tyrosine kinase extensively explored as a target for TKI treatment is the anaplastic lymphoma kinase (ALK). ALK was first described in 1994 as the NPM-ALK fusion protein that is expressed in the majority of anaplastic large-cell lymphomas (ALCL), approximately 55% of adult patients and more than 90% of pediatric patients [3]. There are several reasons why ALK is an ideal target of personalized medicine, including that ALK-transformed cells are in general strongly dependent on ALK tyrosine kinase activity for survival and proliferation and ALK expression is limited in non-tumoral cells, being detected in limited areas of the brain [4]. Therefore, its blockage is catastrophic for cancer cells but irrelevant form normal tissues. Since its discovery, more than 20 different ALK fusion partner genes have been reported across multiple malignancies [5,6]. Perhaps the most widely recognized is the echinoderm microtubule-associated protein-like 4 (EML4)-ALK fusion, identified in 5–6% of non-small-cell lung cancer (NSCLC) patients in 2007 [7]. Even though the relative proportion of NSCLC bearing ALK rearrangements is significantly lower than ALCL or inflammatory myofibroblastic tumors (IMT), ALK-positive NSCLC represent overall the largest cohort of ALK-rearranged patients due the fact that lung cancer has a high incidence worldwide. The identification of ALK rearrangements in lung cancer patients has sparked the development of a series of ALK TKI from different companies. To date, four ALK inhibitors (crizotinib, ceritinib, alectinib and brigatinib) have received approval by the FDA for treatment of ALK-rearranged NSCLC, while others such as lorlatinib have shown promising results in early clinical trials [8]. The use of these new therapies has improved the quality of life and increased the survival of patients, as demonstrated in their respective clinical trials, with remarkable responses in NSCLC patients carrying ALK-rearrangements [9,10,11,12,13,14,15,16]. As with any targeted therapy, ALK-driven NSCLC tumor cells inevitably acquire drug resistance, leading to clinical relapse. At the present time, ALK inhibitors have not yet been approved for use in other ALK-driven cancers than NSCLC; however, some studies have reported remarkable responses, and less frequent relapses, to ALK inhibitors in patients with ALK-positive ALCL and IMT. The apparently higher sensitivity to ALK inhibitors of ALCL and IMT tumors likely reflects a stronger dependency on ALK signaling and/or a lower level of tumor heterogeneity than in ALK-rearranged NSCLC [17,18,19]. Yet, our current knowledge regarding ALK inhibitors resistance originates mostly from ALK-positive NSCLC patients.

While much information has been gathered since the discovery of the first ALK TKI crizotinib to the latest third generation inhibitors regarding the clinical activity of TKIs, there is still limited understanding how acquired resistance develops and undermines the effects of ALK TKIs. This review will summarize the current knowledge about the activity of different ALK inhibitors and their inherent resistance mechanisms that have been reported. We will also discuss potential future therapeutic approaches that can be used to tackle TKI resistance and improve patient outcome.

## 2. Anaplastic Lymphoma Kinase-Physiological Expression and Functional Role

The ALK gene is located on chromosomal region 2p23 and encodes a highly conserved receptor tyrosine kinase (RTK), which is a member of the insulin receptor superfamily, and is most closely related to leukocyte tyrosine kinase (LTK) [20,21,22]. The ALK receptor is composed of an extracellular domain, a single-pass transmembrane region, and an intracellular kinase domain [20]. The extracellular domain contains a glycine-rich region, two MAM segments (meprin, A5 protein, and receptor protein tyrosine phosphatase µ) and one LDLa domain (low density lipoprotein class A). The intracellular portion comprises a juxtamembrane segment, a protein kinase domain and a carboxyterminal tail [6,23,24]. 

The specific role of ALK in human development and physiology is still poorly understood but several studies on different animal models have partially clarified the ALK functions in development. In *Drosophila melanogaster*, ALK signaling is involved in the differentiation of mesenchymal cells, in the development of the visual system [25], the maturation of the neuromuscular junction [26] and in the regulation of body size, learning and memory [27]. In this context, ALK is activated by its ligand Jelly Belly (Jeb) leading to the downstream signaling of the Ras-MAPK pathway [28]. The mammal ALK receptor is unable to bind the Jeb ligand [29], which indicate an evolutionary divergence between mammalians and *D. melanogaster* ALK proteins. In *Caenorhabditis elegans*, SCD-2 (the nematode homolog of ALK), is required for the integration of sensory inputs and the development of neuromuscular junctions [30]. In zebrafish, LTK and ALK show a significant structural homology (such as the presence of MAM domains) and contribute to neural crest nervous system embryogenesis [31]. 

ALK expression patterns throughout the nervous system during mouse embryogenesis suggest important roles in the central nervous system (CNS) development and function in mammals [6,20,32,33]. Iwahara et al. have described that the intensity of ALK mRNA and protein expression in mice diminishes in all tissues after birth, reaching a minimum after three weeks of age and maintained at low levels during the adult life of the animal [20]. Bilsland et al. [34] and Lasek et al. [35], reported that ALK deficient mice are viable and fertile without obvious alterations. Remarkably, the loss of ALK signaling results in a decrease in newborn neurons and in impaired regeneration of myelinated axons [5] and an increased number of progenitor cells within the hippocampus (a defect that can be associated with their behavioral changes) [34]. In 1997, Morris et al. [36] reported that ALK mRNA is expressed in adult human brain, small intestine, testis, prostate, and colon but not in normal human lymphoid cells, spleen, thymus, ovary, heart, placenta, lung, liver, skeletal muscle, kidney, or pancreas. 

Several proteins, such as pleiotrophin (PTN), midkine (MK), osteoblast-specific factor-1 (OSF-1), heparin affinity regulatory peptide (HARP) and heparin-binding neurotrophic factor (HBNF), have been historically reported to be the activating ligands of mammalian ALK [4]. However, recent studies have shown that augmentor α and β (FAM150) are validated ligands of ALK [3,37,38]. Although our knowledge of the mechanism of activation of mammalian ALK protein-tyrosine kinase is incomplete, Lemmon and Schlessinger have described the mechanism of activation of several receptor protein-tyrosine kinases, providing us a hypothetic scheme for ALK activation [21]. Upon ligand binding in the extracellular domain, the receptor protein-tyrosine kinase is activated by inducing receptor dimerization or oligomerization. A possible mechanism for ligand and dimer-induced activation of ALK involves the phosphorylation of one or more of the juxtamembrane tyrosine residues (Tyr 1078, 1092, 1096 and 1131), which in turn would be followed by consecutive phosphorylations until the active form of ALK is established [21]. 

## 3. ALK Gene Alterations in Cancers

The deregulation of tyrosine kinase (TK) activity is one of the major mechanisms of human carcinogenesis and can occur through several mechanisms such as chromosomal translocations, gene amplification or deregulation and point mutation. The abnormal TK activation leads to constitutive activation of several downstream signaling pathways that contribute to the development of neoplastic phenotypes. Tyrosine kinase translocations are found in up to 3% of all human tumors [39]. 

**Figure 1:**
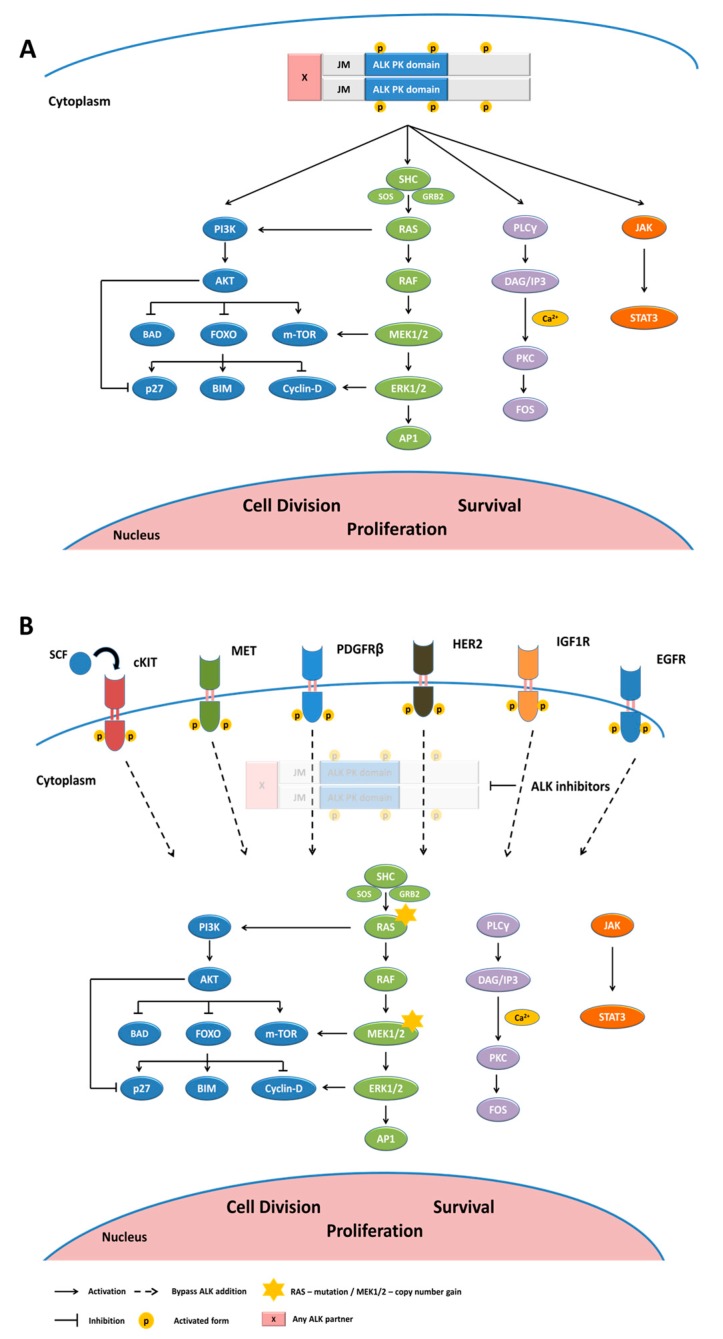
ALK downstream pathways and bypass signaling (**A**) Anaplastic lymphoma kinase (ALK) mediates signaling via the PI3K/AKT, RAS/MAPK, phospholipase Cγ (PLCγ) and Janus kinase (JAK)-signal transducer and activator of transcription (STAT); (**B**) ALK-independent resistance mechanism. Activation of bypass signaling pathways when ALK is inhibited with TKIs: EGFR activation, without EGFR mutations or amplifications; HER2 activation; c-KIT gene amplification in the presence of stem cell factor (SCF); MET activation bypassing ALK inhibitors without anti-MET activity; regulation via transcriptional of PDGFRβ and IGFR activation. Mutations in KRAS and copy number gain of wild-type KRAS; JM, Juxtamembrane.

Usually, translocations comprising transmembrane tyrosine kinase receptors take place between exons that encode the juxtamembrane region or the transmembrane domain. In both cases, these phenomena give rise to the elimination of the extracellular region and, consequently, the ligand-binding regulation, resulting in the constitutive and uncontrolled activation of the fusion typically through an obligatory dimerization dictated by the partner gene [40]. ALK breakpoints are almost invariably located between exons 19 and 20 of ALK. Each translocation creates a fusion protein in which the ALK TK-domain at the 3′-end is connected with distinct proteins portion of different partners at the 5′-end of the fusion, capable of providing constitutive dimerization [41]. ALK rearrangement was first described in 1994, in the anaplastic large cell lymphoma (ALCL) cell lines, with ALK being one of the fused partner in a recurrent chromosomal translocation t(2;5)(p23;q35) together with the nucleophosmin (NPM) gene located on chromosome 5 [36]. This rearrangement produces a fusion gene called NPM-ALK resulting in the expression of an oncogenic fusion protein, NPM-ALK. NPM mediates receptor dimerization of the NPM-ALK protein in a ligand-independent fashion which leads to the constitutive activation of ALK kinase, and ultimately, to the activation of a number of its downstream signaling pathways [20,23,42]. These include JAK/STAT and PI3K/AKT pathways that mediate cell survival and the Ras/Raf/MEK/ERK1/2 pathway which plays a role in cell division and cell proliferation (Figure 1A) [4,6].

Inflammatory myofibroblastic tumor (IMT) was the first non-hematological tumor found to harbor ALK rearrangements in about 50% of cases [43] (Table 1). Non-small-cell lung cancer (NSCLC) was the second non-hematological tumor in which oncogenic *ALK* fusion were detected. In 2007, Simultaneously, Soda et al. and Rikova et al. reported the identification of the EML4-ALK fusion protein in a small cohort of Japanese patients with NSCLC [44]. The novel EML4-ALK fusion protein is the result of an inversion within chromosome 2p that fuses portions of the echinoderm microtubule-associated protein-like 4 (EML4) gene and ALK gene [7]. Since the first report, ALK fusions have been detected in 3% to 7% of NSCLC and associated with a non-smoker history, younger age and adenocarcinoma histology [45]. Many other studies have identified several additional ALK fusion proteins (Table 1) which occur less frequently than EML4-ALK. Moreover, a number of breakpoints variants may be seen for a given fusion protein. EML4-ALK has over 10 distinct variants [46]. Also, it has been reported by Heuckamnn et al. that different ALK fusion genes and EML4-ALK variants exhibited differential sensitivity to crizotinib [47].

With the advent of next-generation sequencing (NSG)-based diagnostics, more than 20 different ALK fusion partners genes have been described in other type of cancer (i.e., colorectal cancer, breast cancer, esophageal cancer, ovarian cancer, renal cell cancer, anaplastic thyroid carcinoma, and diffuse large B-cell lymphoma) even though in low frequencies (Table 1). Armstrong et al. [91] have shown that the level of ALK fusion protein expression and the degree of signaling depend on the partner gene. Using NIH3T3 cells, they were able to demonstrate different effects of ALK fusion proteins on cell proliferation and invasion depending on the exact fusion. In the years following this study, the same group has demonstrated that TPM3-ALK fusion protein expression specifically induces changes in cell morphology and cytoskeleton organization, and it confers higher metastatic capacities than other ALK fusion proteins [92]. 

Additional molecular mechanisms can affect ALK signaling in human cancer other than chromosomal translocations/inversions: ALK up-regulation/amplification and ALK gene mutations [93]. ALK up-regulation has been described in tumors that occasionally harbor ALK-chromosomal translocations, such as NSCLC, rhabdomyosarcoma, breast and ovarian cancer and also reported in neoplasms usually not associated with ALK fusions, such as melanoma, retinoblastoma, Ewing’s sarcoma and neuronal tumors (i.e., glioblastoma, astrocytoma) [4] (Table 1). ALK amplification has also been reported in neuroblastoma almost invariably together with amplification of the adjacent gene MYCN, with possible synergic effects in driving cell growth and survival [94]. ALK TK activation mechanisms in neuroblastoma are not limited to ALK amplification. Mutations in the ALK gene are documented in 4–8% of sporadic neuroblastomas and account for the majority of hereditary cases; ALK variants contribute to the acquisition of neoplastic phenotype and are associated with overall poor-prognosis [94,95]. As observed in ALK rearrangements, ALK point mutations have been described in number of cancers (i.e., anaplastic thyroid cancer [ATC], IMT and NSCLC), although less frequently than in neuroblastoma (Table 1). 

Several studies have permitted the classification of ALK mutations into three different groups: (1) ligand-independent activation mutations; (2) ligand-dependent activating mutations; and (3) kinase-inactivating mutations (known as kinase dead) [96,97]. Ligand-independent mutations (e.g., F1174I, F1174S and F1174L) generate constitutively activated ALK and induce uncontrolled cell proliferation and cell survival [95]; ligand-dependent mutations (e.g., D1091N, T1151M and A1234T) may contribute to pathogenesis [97]. Kinase-inactivating mutations (e.g., I1250T) are very rare and may contribute to the neoplastic phenotype by interfering with the remaining wild-type ALK copy [4]. 

## 4. ALK Inhibitors

### 4.1. Crizotinib: A First-Generation ALK Inhibitor

**Figure 2:**
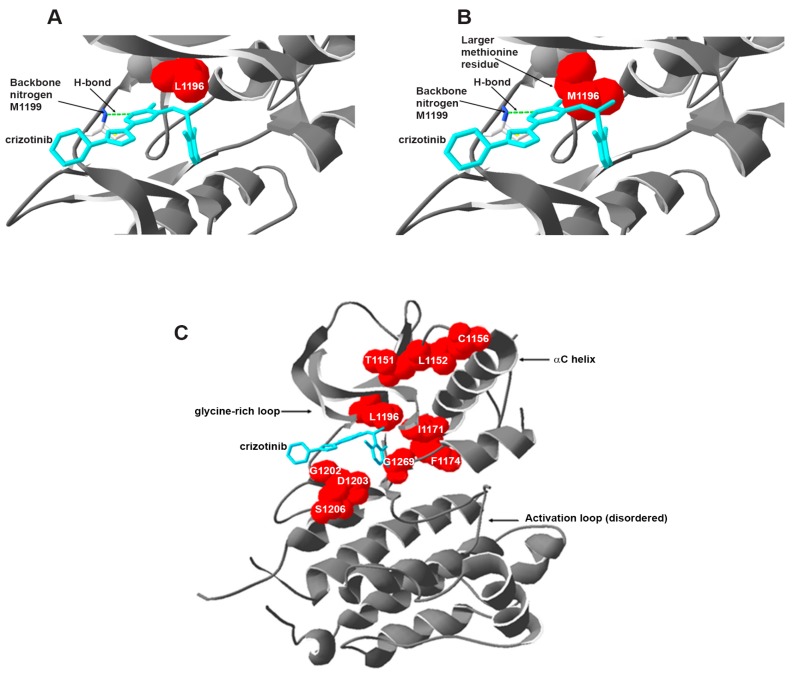
Crystal structure of ALK in complex with crizotinib (PDB: 2XP2). (**A**) Close view of crizotinib bound in the active site of wild-type ALK. The gatekeeper residue L1196 is shown as red surface. Crizotinib is shown as cyan sticks. The green dashed line indicates the hydrogen bonding to the backbone nitrogen of M1199 (indicated in sticks). Secondary structures are represented with grey ribbon; (**B**) The native L1196 from panel A was mutated in silico to M1196, to show steric clash with crizotinib; (**C**) Overall architecture of ALK bound to crizotinib. Key residues associated with resistance to crizotinib are shown as red surface and labeled. Some important regulatory regions of the kinase are indicated by arrows.

Substantial evidence linking aberrations in ALK to various tumors and the success of TKIs such as imatinib and gefitinib led to the discovery and accelerated approval of first ALK inhibitor, crizotinib (PF-02341066 Xalkori). Crizotinib is an orally available drug which was originally discovered as a c-Met kinase inhibitor [98]. The compound binds the ATP pocket of MET kinase in a DFG-in conformation, forming classical hydrogen bonds (Hb) with hinge region residues [99]; in addition, its phenyl ring forms a π-π interaction with the activation loop (A-loop). Ironically, the drug was found to have off-target effects on other kinases including ALK. The crystal structure of crizotinib bound to ALK revealed a similar binding mode, with conserved Hb to the hinge region (Figure 2A), but lacking the π stacking to A-loop, which may explain lower activity against ALK compared to MET. Given the pathogenic role of ALK in different malignancies, crizotinib was then pursued as an ALK inhibitor [100]. Following a number of successful in vitro studies [98] showing the efficacy of crizotinib in ALK inhibition, crizotinib entered into early phase I study (PROFILE 1001) presenting a sustained response in locally advanced or metastatic NSCLC patients carrying the EML4-ALK fusion gene [101]. Subsequently, crizotinib was evaluated in a phase II study (PROFILE 1005) with the final results published recently [102]. 

The objective response rates (ORR) were 54% and 41% in the central and local-testing ALK-detection sub-groups, respectively. Phase II results support the clinical benefits of using crizotinib in ALK-positive NSCLC that had progressed on previous chemotherapy regimens. Two phase III studies, PROFILE 1007 [9] and PROFILE 1014 [10], provided further proof in favor of the use of crizotinib over standard second-line chemotherapy and over first-line chemotherapy, respectively in advanced ALK-positive NSCLC. 

Crizotinib was found to be generally well tolerated in the patients with mostly mild treatment-related adverse events (TRAEs). The most commonly reported TRAEs in ALK-positive NSCLC patients include vision disorder, nausea, diarrhea and vomiting of grade 1 and 2. However, elevated transaminases and neutropenia associated with crizotinib treatment of grade 3 or 4 have also been observed in the patients. Other not so common TRAEs of crizotinib in patients that have been observed over the years, include interstitial lung disease (ILD), bradycardia, QTc prolongation, renal cysts and decreased total testosterone in males. Most of the TRAEs were reversible with crizotinib discontinuation or drug holiday period [102]. Altogether these results led to the approval of crizotinib by FDA for the treatment of locally advanced or metastatic ALK-positive NSCLC in 2011. Additionally, significant therapeutic responses have also been reported in ALCL [103,104], neuroblastoma [105], and IMT [18] patients. There are ongoing clinical trials that are evaluating long-term efficacy and safety profile of crizotinib in patients carrying ALK gene abnormalities.

### 4.2. Second Generation ALK Inhibitors

Even though there are diverse mechanisms through which resistance against ALK inhibition has been shown to develop, crizotinib-resistant tumors still continue to be ALK-dependent for their growth in many cases. Around 30% of crizotinib-resistant NSCLC patients develop secondary resistance mutations in the ALK TK domain [46]. Therefore, more potent, selective and structurally different next-generation ALK inhibitors have been developed or are in the pipeline to overcome crizotinib resistance. Although they are not functionally or structurally related to crizotinib (except lorlatinib, see below) they are usually referred as second-generation inhibitors, as they were all developed to tackle crizotinib-resistance mutants. Eight novel ALK inhibitors have entered the clinic, including ceritinib, alectinib, and brigatinib, that have demonstrated potent and durable activity in ALK-positive NSCLC.

#### 4.2.1. Ceritinib (LDK378; Zykadia; Novartis)

Ceritinib is an ATP-competitive, selective oral ALK inhibitor that was found to be 20 fold more potent than crizotinib in enzymatic assays [106,107]. It was developed starting from the original first-generation, non-clinical compound NVP-TAE684 [108] with a few significant structural changes, in order to increase kinase selectivity and reduce the formation of reactive metabolites that impaired NVP-TAE684 clinical development due to toxicity [106]. The new compound (LDK378) was shown to form reactive adducts in negligible amount compared to its parent compound, while maintaining low nanomolar anti-ALK activity. Ceritinib also showed activity against insulin-like growth factor 1 receptor (IGF-R1), insulin receptor (IR) and ROS1 but with a 5–11 fold higher IC50 as compared to its IC50 for ALK. Ceritinib inhibited in vitro and in vivo the growth of ALK-positive cells carrying crizotinib-resistant mutations, L1196M, G1269A, I1171T, and S1206Y but failed to inhibit the growth of G1202R and F1174V/C mutants [107]. Structural data can explain why ceritinib retains potency against some crizotinib-resistant mutants: for instance, while mutation of Gly1269 to Ala causes steric clash with the halogenated phenyl ring of crizotinib, it is not predicted to have any impact on ceritinib binding. Similarly, ceritinb interacts equally well with Leu1196 as with Met1196 [107]. Phase I study, conducted on ALK+ NSCLC patients that had been previously treated with cytotoxic chemotherapy or crizotinib, showed an ORR of 58% in patients who received ceritinib at a daily dose of 750 mg [11]. Based on the pre-clinical studies and ASCEND-1 data, ceritinib received an accelerated approval from FDA for the treatment of ALK-positive metastatic NSCLC patients with disease progression or intolerance to crizotinib. Subsequently, ceritinib demonstrated higher anti-tumor efficacy in ALK-rearranged NSCLC patients previously treated with chemotherapy and crizotinib as well as in crizotinib naïve patients during ASCEND-2 [109] and ASCEND-3 [110] clinical trials. Results from the ASCEND-4, a randomized, open-label, phase 3 study, were published recently [111]. The study evaluated the efficacy and safety of ceritinib in comparison to platinum-based chemotherapy as a first line treatment in advanced ALK-rearranged NSCLC. The median progression-free survival of ceritinib-treated group was 16.6 months as compared to 8.1 months in the chemotherapy-treated group. Most of the adverse events related to ceritinib treatment reported in the study were of grade 1 or 2 gastrointestinal (GI) toxicity (diarrhea, nausea, vomiting) and grade 3 or 4 hepatictoxicity (increased alanine and aspartate aminotransferases). 80% of the patients needed dose reduction or interruption to manage these adverse events [111]. Ceritinib is approved at 750 mg per day in a fasted state for expanded use in first-line ALK-positive metastatic NSCLC [112]. Since most of the serious adverse events (SAE) to ceritinib treatment are GI toxicity related, a multicenter, randomized open-label study ASCEND-8 evaluated the safety profile of ceritinib at lower doses (450 mg or 600 mg) taken daily with a low-fat meal compared to 750 mg daily in fasted patients with ALK-positive NSCLC [113]. Results from the study show that a lower dose of ceritinib (450 mg) taken with food reduced the number of GI toxicity related AE. Most of the GI toxicities in the 450 mg dose arm were mostly grade 1, and no grade 3 or 4 GI toxicities were reported in that arm. Additionally, the number of patients requiring dose adjustment or drug interruption in the 450 mg ceritinib arm were the lowest compared to the 600 mg with food ceritinib and 700 mg fasted ceritinib treatment arm [113]. These results indicate that a lower dosage of 450 mg ceritinib taken with food maintains the same exposure as the currently approved dose of 750 mg fasted but with less severe and frequent GI toxicity profile.

#### 4.2.2. Alectinib (CH5424802; Chugai-Roche)

Alectinib is another second generation ALK inhibitor, highly selective and potent against the ALK tyrosine kinase protein [114]. It binds the ATP binding site of ALK, forming a canonical Hb with M1199. In addition, alectinib interacts via solvent water molecules with several other surrounding residues from the αC-helix (K1150, E1167), the catalytic loop (R1253) and the DFG motif (G1269, D1270). The compound is thus embedded in a stabilizing global Hb network which can probably compensate for any single mutation at the binding site. Moreover, alectinib establishes a π interaction with L1196, which is maintained when Leu is mutated to Met, accounting for its high activity against the crizotinib-resistant gatekeeper L1196M mutant [115]. A phase II study in Japan reported an ORR of 93.5% with alectinib treatment in ALK+ NSCLC patients who had not been treated with an ALK inhibitor [116]. Apart from its excellent activity against ALK, alectinib also showed remarkable activity in patients with CNS metastases [117]. Alectinib received a breakthrough therapy designation (BTD) by the FDA for ALK-positive NSCLC patients who progressed on crizotinib while it was approved in Japan in 2014 for the treatment of ALK-rearranged NSCLC patients. Alectinib also showed substantial efficacy against crizotinib-resistant and/or ALK secondary mutations including the gatekeeper L1196M in vitro and in vivo [115,118] however, it was less effective against the G1202R [118]. Additionally, other ALK resistance mutations (V1180L, I1171T, F1174V) have been observed that arise against alectinib treatment [119]. Two phase II studies, the North American study (NCT01871805) and global study (NCT01801111), evaluated the safety and efficacy of alectinib in 87 and 138 ALK+ NSCLC patients who had progressed on crizotinib, respectively [14]. The patients received alectinib at a dose of 600 mg BID. ORR of 48% and 50% were reported recently from the North American and global study respectively. Most common side effects reported in the studies were constipation, fatigue, myalgia and peripheral edema. Grade 3 or higher AEs were observed in 26% of patients that included increased blood creatine phosphokinase and neutropenia [120]. In the global phase II study, the CNS ORR with baseline measurable CNS lesions was 57% while in the North American study the intracranial response was reported to be 75%. These two studies have demonstrated that alectinib is effective and well tolerated in ALK+ NSCLC patients refractory to crizotinib. Results from a randomized phase III trial comparing alectinib with crizotinib in treatment naïve ALK+ NSCLC has been published recently. Peters S et al. have showed that alectinib was more efficacious and less toxic as a primary treatment for the patients [15].

#### 4.2.3. Brigatinib (AP26113; Ariad)

Brigatinib, another orally available potent next-generation-ALK/ROS1/EGFR inhibitor had displayed activity against the tyrosine kinases as well as some of their mutant forms in cellular and pre-clinical models [121]. Brigatinib is a close analogue of NVP-TAE684, with the original sulfonyl group replaced by a phosphine-oxide moiety. According to structure-activity relationship (SAR) data, this group confers favorable Absorption, Distribution, Metabolism, and Excretion (ADME) properties to the molecule and higher selectivity versus IGF1R and IR [121]. Similar to other ALK inhibitors, brigatinib forms Hb to the hinge region residue L1198 as well as the gatekeeper L1196. Preclinical data showed that brigatinib has pan-ALK inhibitory profile (i.e., blocks all crizotinib-resistant mutants) in cellular models at clinically achievable levels [122], although it still suffers a significant loss of activity against the G1202R mutant [123,124]. A phase I/II study to evaluate the safety and activity of brigatinib was recently reported [125]. Phase I study aimed to establish the recommended phase II dose of brigatinib in patients with advanced malignancies other than leukemia. Based on phase I results, three regimens were tested in the phase II: 180 mg daily, 90 mg daily and 180 mg daily with a 7-day lead-in at 90 mg daily. The phase II expansion study was divided into five histologically and molecularly defined cohorts based on prior chemotherapy and/or tyrosine kinase inhibitor treatments as well as the cancer types and CNS involvement. Crizotinib pre-treated ALK-rearranged NSCLC patient cohort had a confirmed objective response of 62% with a median progression-free survival of 13.2 months upon brigatinib treatment. Kim et al. have published results from the ongoing phase II, randomized, open-label, multicenter international study (ALK in Lung Cancer Trial of brigatinib; ALTA, ClinicalTrials.gov identifier: NCT02094573) that evaluated the efficacy and safety of two different brigatinib dosage regimens (90 mg daily and 180 mg daily) in crizotinib-treated ALK+ locally advanced or metastatic NSCLC patients [16]. After a median follow-up of 8 months, investigator-assessed ORR was 45% and 54% in the 90 mg daily and 180 mg daily dosage groups, respectively. A confirmed partial response in a patient with the G1202R mutation was also reported from the 180 mg daily group. In the phase II study, most common treatment-emergent adverse events (TEAE) included GI symptoms, headache and cough that were of low grade. AEs of grade >3 were hypertension, increased blood creatine phosphokinase, pneumonia and increased lipase. Pulmonary AEs (dyspnea, hypoxia, cough, pneumonia, and pneumonitis) with an early onset, usually within 24–48 h of treatment initiation, were observed in phase I/II study as well as in the phase II study [16,125]. In the ALTA study, all the pulmonary AEs occurred only at 90 mg brigatinib dose while no such events occurred after escalation to 180 mg dose [119]. On 28 April, 2017, the FDA granted an accelerated approval to brigatinib for the treatment of ALK+ metastatic NSCLC patients [126]. A phase III trial, ALTA-1L (NCT02737501) is ongoing to compare the efficacy and safety of brigatinib with those of crizotinib as a first-line treatment in patients with ALK+ metastatic NSCLC.

### 4.3. Other ALK TKI Under Development

Apart from the above mentioned inhibitors, there are other tyrosine kinase inhibitors that are under development (pre-clinical or clinical). Table 2 lists currently available details regarding these small molecule inhibitors. Given the developing resistance against the second-generation inhibitors, these new inhibitors are anticipated to be more potent against ALK-driven tumor cells, have improved intracranial penetrance as well the ability to overcome the existing drug resistance. One example is lorlatinib (PF-06463922), a potent and brain-penetrant third generation TKI developed by Pfizer through cyclization and further modification of their first-generation compound, crizotinib, with the aim to improve brain penetration and inhibition of drug-resistant ALK mutants [127]. Indeed, lorlatinib showed good brain exposure and broad activity against resistant mutations. The compound interacts with the P-loop (L1122, G1123, and V1130) and with the conserved K1150. Lorlatinib inhibited wilt-type and mutant ALK at a sub-nanomolar concentrations in cell-line models. It also exhibited high potency against all known clinically acquired ALK mutations, including the highly resistant G1202R mutant [128]. Zou HY et al. have demonstrated in in vivo experiments, that lorlatinib lead to regression of EML4-ALK-driven brain metastases ensuring a prolonged mouse survival [128]. In a phase I study, 42% (11/26) ALK-positive NSCLC patients who had been previously treated and progressed on first- and second-generation ALK TKIs, responded to lorlatinib. Also, lorlatinib showed both systemic and intracranial activity. These results suggest that lorlatinib may be an effective therapeutic approach for patients with ALK-driven NSCLC who have become resistant to the currently available TKIs, including second-generation ALK TKIs [8].

In the phase I, dose escalation study, commonly observed AEs were hypercholesterolemia, hypertriglyceridemia, peripheral neuropathy, and peripheral edema in 72%, 39%, 39%, and 39% patients, respectively. GI symptoms (constipation and nausea) were less frequent and predominantly grade 1. The authors also reported mild neurocognitive side-effects (difficulty multitasking, slowing of speech, and short-term memory deficits) and mood side-effects that were reversible with dose interruption or dose reduction. However, how lorlatinib affects the lipid metabolism and causes hypercholesterolemia, hypertriglyceridemia, AEs unique to lorlatinib treatment, is still not known [8]. A phase III study comparing lorlatinib with crizotinib as monotherapy in terms of prolonging progression-free survival and overall survival in treatment naïve advanced ALK-positive NSCLC patients is currently ongoing (NCT03052608). Even though lorlatinib is a potent inhibitor, the L1198F resistant mutation was reported in one ALK+ NSCLC patient after receiving lorlatinib treatment for 8 months [147]. The patient had been treated with two prior TKIs; crizotinib and ceritinib and became refractory to both of them. Surprisingly, the L1198F lorlatinib resistant tumor regained sensitivity to crizotinib [147].

## 5. ALK TKI Resistance Mechanisms

Resistance to targeted therapies can be either primary or acquired. Primary resistance to a targeted therapy implies an intrinsic lack of response to the treatment from the beginning while acquired resistance denotes disease progression after an initial response (partial or complete) to the therapy [120]. Though mechanisms of intrinsic resistance are poorly understood, acquired resistance mechanisms broadly fall under two categories; ALK-dependent or ALK-independent mechanisms of resistance.

### 5.1. ALK-Dependent Resistance Mechanisms

#### 5.1.1. Secondary Mutations in the ALK Tyrosine Kinase Domain

In general, secondary mutations within the target kinase cause drug resistance by re-activation of the kinase and its downstream signaling pathways despite the presence of the TKI. These resistance mutations often occur around the surface lining the drug binding site (Figure 2C), although a number of mutations have been described that lie far from the active site. Depending on their location, mutations can directly hamper TKI binding to the target kinase, alter the conformation of the kinase, and/or modify the ATP-binding affinity of the kinase. 

##### Resistance against Crizotinib

Despite the remarkable responses that have been observed in patients with ALK rearrangements, resistance to crizotinib eventually develops and rather quickly, making durable response unachievable, particularly in NSCLC. One of the important mechanisms of acquired resistance to crizotinib is the selection of point mutations within the drug target that alter drug sensitivity (Table 3). The first case of resistance against crizotinib was reported in an EML4-ALK-positive NSCLC patient [148]. The tumor resumed growth after an initial partial response over a period of 5 months. Deep sequencing analysis of the patient sample revealed a L1196M mutation and a C1156Y substitution at a relatively high frequency. The L1196 residue is a conserved gatekeeper residue located close to the ATP pocket and crizotinib binding site. In this secondary mutation, a smaller residue (leucine) is replaced by a larger residue (methionine) (Figure 2A,B). In contrast to a larger residue, a smaller one does not block the access of the inhibitor to the adjacent hydrophobic pocket [149]. Methionine substitution, in addition, has been reported to increase the enzyme activity by strengthening the hydrophobic R-spine which then promotes the formation of the active protein conformation [150]. L1196M mutant EML4-ALK protein was found to have higher phosphorylation levels [142]. These results show that the L1196M substitution confers drug resistance by increasing the protein kinase activity. On the other hand, the C1156Y mutation creates a displacement of crizotinib along with some conformational changes in the binding site of the drug that eventually decreases crizotinib affinity and leads to drug resistance [151]. Interestingly, a different gatekeeper mutation (L1196Q) was identified in crizotinib-resistant ALCL cells in vitro [152]. The same paper described an I1171N mutant that was resistant to all tested inhibitors; this mutation was later identified in an ALCL patient progressing on crizotinib [17]. Sasaki and colleagues described another case of crizotinib resistance in an IMT patient [153]. These investigators found the F1174L mutation in the RANBP2-ALK kinase domain in the relapsed tumor lesions. The F1174L mutation had earlier been detected in neuroblastoma [95]. The 1174 residue is found at the carboxyterminal end of the αC-helix and has been shown to reduce ALK sensitivity to crizotinib by increasing ATP binding affinity in neuroblastoma cell lines and in vivo models [154]. Another mutant variant at the same position, F1174V, was also found in an ALK+ NSCLC patient resistant to crizotinib [155]. Secondary mutation L1152R with an EGFR and c-Met hyperactivation was reported in a cell line established from the NSCLC patient who relapsed after 3 months of crizotinib treatment [156]. The L1152R mutation affected crizotinib-mediated inhibition of downstream AKT and ERK phosphorylation in the resistant cells. As the L1152R mutation does not seem to be in direct contact with the ATP-binding pocket [157], how L1152R mediates ALK inhibitor resistance is still unclear. A number of other secondary mutations such as S1206Y, G1202R, 1151Tins, G1269A were also found in crizotinib-refractory NSCLC patients (Figure 2C) [158,159]. Both, G1202R and S1206Y, are located at the solvent front of the kinase domain and presumably interfere with inhibitor binding due to steric hindrance and conformational changes of the kinase. While the insertion of a threonine residue at 1151 position is speculated to lead to a change in the affinity of ALK for ATP [157]. The Gly1269 residue is situated at the end of the ATP-binding pocket of ALK and its substitution with the larger Ala residue leads to a decrease in the binding of crizotinib to ALK due to steric hindrance [159]. Another ALK mutation found commonly in neuroblastoma is R1275Q [94], which has been shown to increase the ATP-binding affinity in the mutated ALK in vitro [154]. 

##### Resistance to Second-Generation ALK TKIs

Even though the second generation of ALK inhibitors is proven to be more potent and highly selective with tolerable adverse events, the biggest setback still stays in the form of acquired resistance against them. For example, while ceritinib was able to overcome some of the secondary ALK resistance mutations that arise after crizotinib treatment, G1202R, F1174C/V mutations were reported to be selected by ceritinib. Structural analysis revealed that G1202R substitution causes a significant loss in ceritinib binding due to steric hindrance [107]. Other secondary mutations such as C1156Y, 1152Tins, and L1152R, G1123S have also been documented to be associated with resistance against ceritinib [160,168].

On the other hand, alectinib was shown to be effective against crizotinib or ceritinib resistant mutations, but leads to the acquisition of I1171T and V1180L resistant mutations in vitro and in a patient upon alectinib treatment. Interestingly, these two mutations could be overcome with ceritinib treatment which supports the idea of using two different inhibitors/combinatorial therapy. Again, the G1202R emerged as a highly intractable mutant [169]. Indeed, this mutation was reported to be resistant to all clinically available inhibitors, thereby representing the biggest current clinical challenge [123]. Point mutations L1122V, F1174V+L1198F, S1206C, and L1198F have been shown to confer resistance against brigatinib in ALCL cell lines [166]. Except for the S1206C mutation, most of the brigatinib resistance could be overcome by switching back to crizotinib, other ALK TKIs or using alternative inhibitors such as heat shock protein 90 (HSP90) inhibitors [166]. 

Emergence of compound mutations upon sequential TKI treatment appears to be the next hurdle. Given the structural differences among the available ALK TKIs, it is perhaps not surprising that each ALK TKI appears to be associated with a specific profile of secondary ALK resistance mutations (Table 3). One such example of the compound mutations is the presence of a double mutation, C1156Y and L1198F in an advanced ALK+ NSCLC patient treated sequentially with crizotinib, ceritinib and lorlatinib [147]. Even though C1156Y mutation is sensitive to lorlatinib, the addition of L1198F disrupts binding of the drug with the kinase and leads to lorlatinib resistance. But interestingly, in vitro studies showed that L1198F mutation paradoxically leads to re-sensitization to the less potent and selective inhibitor crizotinib. Based on these findings, the patient was retreated with crizotinib and had a durable response [170]. Other examples of the compound mutation phenomenon include detection of C1156Y and I1171N double mutation after progression on crizotinib, ceritinib, and alectinib sequential treatment and presence of E1210K with D1203N mutation after sequential crizotinib and brigatinib treatment [129]. Given the number of different ALK TKIs that are being approved and their implementation in clinic for sequential TKI treatment, we are bound to see an increase in the number and variety of compound mutations (Table 3). 

#### 5.1.2. Amplification of ALK

Another ALK-dependent resistance mechanism is the amplification of *ALK* gene which occurs less frequently than secondary mutations, but is a recognized cause of acquired resistance to crizotinib. Katayama et al. reported high-level of wild type EML4-ALK gene amplification in 1 of the 15 patients that progressed on crizotinib [158]. The authors did not find any additional secondary mutations in the sample. Doebele et al. also documented an increase in the copy number of rearranged *ALK* gene per cells in 2 out of 12 patients’ samples from post-crizotinib treatment [159]. Copy number gain (CNG) in the rearranged *ALK* gene was accompanied by the resistant mutation G1269A in 1 of these 2 samples. Based on the present clinical evidence it is difficult to say under which circumstances/factors, amplification of *ALK* gene is sufficient enough to render the tumor cells resistant. Genomic amplification of ALK locus has also been described to mediate ALK TKI resistance in ALCL cell lines [166,171]. Ceccon et al. observed that the brigatinib resistant ALCL cells had overexpressed NPM-ALK due to the ALK amplification [166]. Interestingly, the resistant cells were dependent/addicted to the TKI for their growth and proliferation [172]. Remarkably, drug withdrawal lead to apoptotic death of these drug-addicted TKI resistant cells mediated by the activation of the DNA damage response pathway due to an unbalanced NPM-ALK signaling [172].

### 5.2. ALK-Independent Resistance Mechanisms

#### 5.2.1. Activation of Bypass Signaling Pathways

One important category of ALK-independent resistance mechanism is the activation of bypass signaling pathways through genetic alterations, autocrine signaling, or dysregulation of feedback signaling which leads to the survival and growth of tumor cells even when the target driven gene is inhibited with the TKI.

One such example is the epidermal growth factor receptor (EGFR) activation [156,158,173]. Studies conducted in ALK-rearranged lung cancer cell lines have shown an increment of EGFR phosphorylation in crizotinib-resistant cell lines which did not present secondary ALK mutation/up-regulation, when compared with parental crizotinib-sensitive cells, leading to a persistent activation of downstream ERK and AKT signaling. However, those cells did not present any EGFR mutations or amplification, telling that EGFR activity may result from receptor or ligand up-regulation [156,173]. Gene expression profiling of crizotinib-resistant versus crizotinib-naive NSCLC tumor samples using RNA sequencing followed by single-sample gene set enrichment analysis (ssGSEA) has identified EGFR and HER2 (members of the HER receptor family) signatures as two of the most enriched gene expression marks in resistant tumors [174]. 

In ALK-positive lung adenocarcinoma cell lines and mouse xenograft models, the RAS–MEK pathway was found to be the critical downstream effector of EML4–ALK. In a recent study, using next generation sequencing analysis in a patient-derived ALK-translocated lung cancer cell line after ceritinib treatment, a MAP2K1-K57N activating mutation was found as the primary genetic alteration which was leading to MEK activation. More importantly, a separate study verified that ALK/MEK dual blockade may be effective not only in overcoming but also in delaying ALK TKI resistance [175,176]. In addition, c-KIT gene amplification in the presence of stem cell factor (SCF) has also been reported to impart some degree of resistance against crizotinib in patient samples [158]. A combination of crizotinib and imatinib (c-KIT/ABL inhibitor) treatment was able to overcome the resistance in c-KIT overexpressing crizotinib-resistant H3122 cells [158]. 

Laimer et al. [117] have shown in a mouse model of NPM-ALK-triggered lymphomagenesis, that the activator protein 1 family members JUN and JUNB promote lymphoma development and tumor dissemination via transcriptional regulation of platelet-derived growth factor receptor-β (PDGFRβ). When PDGFRβ is inhibited therapeutically, the survival of NPM-ALK transgenic mice is prolonged. Also, its inhibition leads to an increased efficacy of an ALK-specific inhibitor in transplanted NPM-ALK tumors. Remarkably, a patient with refractory late-stage ALK-rearranged ALCL treated with PDGFRα and PDGFRβ inhibitors had a rapid and complete remission [177]. 

Doebele et al. reported mutation in *KRAS* gene in 2 of the 11 NSCLC patients who relapsed on crizotinib [159]. One patient had a KRASG12C mutation which was detected in both, pre- and post-crizotinib biopsy samples. The second patient had a G12V substitution in *KRAS* gene only in the post-crizotinib biopsy sample. Interestingly, when the author introduced the G12V substitution in H3122 cells to evaluate its effect on resistance, they did not see a significant difference in IC50 values between parental and mutant cells [159]. Additionally, re-activation of MAPK signaling pathway due to a copy number gain (CNG) of wild-type *KRAS* gene or reduced levels of MAPK phosphatase DUSP6 was also reported to impart resistance against ALK TKIs in mouse models [175]. Using an upfront dual ALK and MEK-inhibitor therapy the authors were able to suppress the development of resistance in vitro. Other examples of bypass mechanisms clinically implicated in ALK TKI resistance include PIK3CA mutations (one of 27 samples (3.7%), post-alectinib; a case post-ceritinib) [129,176], IGF1R activation (four of five samples (80%), post-crizotinib [178]; and SRC activation [176]) (Figure 1B).

#### 5.2.3. Other Mechanisms

In case of NSCLC, change in morphology has also been shown to contribute towards TKI resistance. Epithelial-to-mesenchymal transition (EMT) is one such morphological change in which epithelial cells lose their polarity and cell-to-cell junction and become more fibroblastic as well as more motile and invasive. EMT has been reported to confer resistance against first and second generation ALK TKIs in NSCLC cell lines [179] as well tumor samples [129]. However, how exactly and to what extent EMT contributes to this resistance still needs to be uncovered. Another recently identified mechanism of resistance against ALK TKIs is the histological transformation from a NSCLC entity to Small Cell Lung Cancer (SCLC). Several reports have been published reporting the transformation in NSCLC patients after progression on crizotinib [180,181], alectinib [182,183,184] and also on ceritinib [185]. In all of these reported cases, the SCLC tumor cells retained the ALK expression. None of the investigators were able to firmly demonstrate if the SCLC transformation appeared as a novel resistance mechanism or the SCLC cells could have co-existed but not discovered during the initial diagnoses. Even though the transformation mechanism is not yet completely understood, loss of retinoblastoma (*RB*) gene seems to be important for this type of transformation. Mutations in *TP53* and *PTEN* genes have also been found in a patient presenting with the SCLC transformation [185]. 

## 6. Other Therapeutic Strategies to Overcome ALK-Related Resistance

### 6.1. ALK TKIs Combined with Other Inhibitors Targeting Different Kinases

The majority of studies performed on ALK TKI resistance has focused on the development of next-generation ALK inhibitors, which can overcome at least some of resistant mutants. Around 30% of crizotinib resistance in ALK-positive NSCLC is related to secondary ALK mutations and/or amplifications, which maintain their sensitivity to next-generation ALK inhibitors. However, nearly to 40% of the resistant cases to second-generation inhibitors is no longer ALK-dependent. Activation of bypass signaling has emerged as other potential strategy to combat ALK TKI resistance. Combination strategies that target both ALK and a second kinase may be needed to overcome the different bypass pathways that mediate ALK resistance. 

As mentioned above, MEK reactivation is a key example of resistance mechanism involving other TKs. Crystal et al. in a patient-derived ALK-rearranged lung cancer cell line post-ceritinib harboring MAP2K1^K57N^ activation mutation of MEK, have shown that the MEK inhibitor selumetinib was a potent hit when combined with ceritinib [176]. Confirming these results, a separated study lead by Hrustanovic et al. demonstrated that the dual blockage of ALK/MEK may be effective not only in overcoming but also in delaying ALK TKI resistance [175]. Based on these findings, a large variety of combination therapies of ALK and MEK inhibitors may be a potential therapeutic strategy.

Current clinical trials are testing the efficacy of ALK TKI in combination with other target agents. Alectinib combined with bevacizumab (angiogenesis agent targeting vascular endothelial growth factor–VEGF) is being tested in patients with ALK-rearranged NSCLC with at least one target lesion in CNS (NCT02521051) and combinations of ceritinib with either LEE011 (CDK4/6 inhibitor) or everolimus (mTOR inhibitor) are in early-phase testing in NSCLC (NCT02292550 and NCT02321501, respectively). Other potential combinations include ALK TKI with EGFR inhibitor, cKIT inhibitor and SRC inhibitor. The selection of the appropriate combination should be individualized based on the resistance mechanism identified and toxicities of combinations may be a major limitation. MET activation is a very well-known bypass signaling pathway in EGFR-mutant NSCLC but in ALK-rearranged NSCLC does not cause resistance to the first-generation TKI crizotinib, which is also a potent MET inhibitor [186,187]. However, some of the next-generation ALK TKIs do not have activity against MET and indeed, MET has been reported as a bypass signaling mechanism in a patient who has failed second-generation ALK inhibitors. This patient subsequently responded to crizotinib [188]. Similarly, the fact that each ALK TKI is associated with a unique spectrum of ALK resistance mutations, suggests that combinations of ALK TKIs could also be beneficial and enable more durable responses than those achieved in monotherapies. 

### 6.2. ALK Inhibitors Combined with Immunotherapy

#### 6.2.1. Immune Checkpoint Inhibitors 

Immunotherapy with immune checkpoints inhibitors, specifically PD-1 and PD-L1, has demonstrated good responses in advanced NSCLC, ranging from 15 to 20%, with some patients exhibiting durable responses after discontinuing therapy [189,190,191,192,193,194]. In 2015, two immune checkpoints inhibitors received FDA approval for second-line therapy of NSCLC, namely nivolumab and pembrolizumab both targeting the programmed cell death-1 (PD-1). In 2016, another checkpoint inhibitor (atezolizumab, a programmed cell death-ligand 1 [PD-L1]) received its approval from FDA for first-line NSCLC treatment in patients with high PD-L1 expressing tumors [189,195,196,197,198]. Some clinical trials are investigating the efficacy and safety of an ALK TKI combined with immunotherapy in lung cancer, namely, crizotinib with nivolumab or ipilimumab (NCT01998126) or pembrolizumab (NCT02511184); alectinib with atezolizumab (NCT02013219); ceritinib combined with nivolumab (NCT02393625) and lorlatinib with avelumab (NCT02584634). Yet, there are still limited preclinical data to support this combination strategy. Although those immune checkpoints inhibitors have demonstrated durable responses after disruption of the therapy, only 20% of the patients benefit this effect and it has been associated with high expression levels of PD-L1, high mutational load and smoking history [189,191]. Patients bearing ALK-rearrangements tend to be never-smokers and with a low tumor mutational load [199] and to respond poorly to PD-1 blockade [200]. Thus, the potential benefit of the addition of immunotherapy to ALK TKI treatment is still unclear.

#### 6.2.2. Vaccine Therapy

The administration of immunogenic tumor-associated antigens or cells in conjunction with an immune-adjuvant that elicits specific antitumor immune response, boost the immune system against tumor cells [201]. These therapeutic vaccines identify specific tumor-associated antigen and elicit the immune system against them. ALK has unique biological characteristics that are attractive for a tumor antigen. First, ALK is not expressed in obviously detectable levels by non-tumoral cells with the exception of specific regions of the central nervous system and the testis, both immunologically privileged sites. Many evidences support that ALK is spontaneously recognized as a tumor antigen in human patients [202]. Circulating antibodies against NPM-ALK and EML4-ALK proteins were found in ALK-positive ACLC and NSCLC, respectively [203,204]. In 2008 we have demonstrated in vivo the efficacy of a DNA-based vaccine encoding portions of the cytoplasmic domain of ALK. This combination enhanced the survival of mice challenged with ALK-positive lymphomas [205]. More recently, our group has shown that ALK vaccination induce a strong and specific immune response either prophylactically or therapeutically against ALK+ lung tumors in preclinical models. The ALK vaccine in combination with ALK TKI treatment significantly delayed tumor relapse after TKI suspension [206]. Many of the vaccine trials in NSCLC showed an immune response after vaccination, usually in form of an increase of target specific cytotoxic T-cells. Unfortunately, this has not translated into significant survival advantage in the phase III trials to date. In terms of toxicity, most of these vaccine-based therapies show less toxicity when compared to traditional chemotherapies or other immune therapies. While vaccine therapy trials in NSCLC have so far failed to show significant clinical benefit, the demonstration of enhanced immune response in these trials suggests that an ALK-directed vaccine therapy could have more degree of clinical efficacy in combination with checkpoint inhibitors.

## 7. Conclusions

ALK represents a validated therapeutic target in numerous malignancies such as NSCLC, ALCL, IMT and neuroblastoma. Since its discovery as a fusion oncogene, four ALK inhibitors have been approved and will become standard of cure for NSCLC patients harboring ALK-rearrangements. More ALK inhibitors are in clinical development and some have already shown strong efficacy in cohorts of patients with ALK-positive tumors. Notwithstanding these remarkable responses, ALK TKIs effect is transient and never achieves a complete cure. Patients invariably relapse due to acquired resistance, which represents a significant barrier to the successful treatment of ALK-positive patients. Therefore, the development of strategies to overcome/prevent/delay resistance is a priority. With the current knowledge of the complex and heterogeneous mechanisms process behind ALK resistance, multiple next-generation ALK inhibitors and combinatorial treatment approaches can be envisioned. These potential new therapeutic strategies have the promise to improve the treatment of an increasing portion of patients ALK-positive cancers.

## Figures and Tables

**Table 1:** ALK rearrangements in human malignancies.

Cancer Type	ALK Fusion Partner (Chromosomal Localization)	Frequency %	References
ALCL	NPM1 (5q35.1) TPM3 (1q21.3) ATIC (2q35) TFG (3q12.2) TRAF1 (9q33.2) CLTC (17q23.1) RNF213 (17q25.3) TPM4 (19p13.1) MYH9 (22q12.3) MSN (Xq12) Aditional rare rearrangements	~55% (in adults)	[36,48,49,50,51,52,53,54,55,56,57]
Breast cancer	EML4 (2p21)	N.D.	[58]
Colorectal cancer	EML4 (2p21) WDCP (2p23.3)	<1%	[58,59,60,61]
DLBCL	RANBP2 (2q13) EML4 (2p21) SEC31A (4q21.22) SQSTM1 (5q35) NPM1 (5q35.1)	<1%	[62,63,64,65,66,67,68]
Esophageal cancer	TPM4 (19p13.1)	N.D.	[69,70]
IMT	TPM3 (1q21.3) RANBP2 (2q13) ATIC (2q35) SEC31A (4q21.22) CARS (11p15.4) PPFIBP1 (12p11) CLTC (17q23.1) TPM4 (19p13.1)	Up to 50%	[43,49,71,72,73,74,75,76,77,78,79,80]
NSCLC	EML4 (2p21) TPR (1q31.1) CRIM1 (2p22.2) STRN (2p22.1) TFG (3q12.2) HIP1 (7q11.23) PTPN3 (9q31) KIF5B (10p11.22) KLC1 (14q32.3) CLTC (17q23.1)	3–7%	[7,44,81,82,83,84]
Ovarian cancer	FN1 (2q35)	N.D.	[85]
RCC	VCL (10q22.2) TPM3 (1q21.2) EML4 (2p21) STRN (2p22.2)	<1%	[86,87,88,89]
RMC	VCL (10q22.2)	N.D.	[90]

Abbreviations (alphabetic order): ALK, anaplastic lymphoma kinase; ALCL, anaplastic large-cell lymphoma; ATIC, 5-Aminoimidazole-4-Carboxamide Ribonucleotide Formyltransferase/IMP Cyclohydrolase; CARS, cysteinyl-tRNA synthetase; CLTC, clatherin heavy chain; CRIM1, cysteine rich transmembrane BMP regulator 1; DLBCL, diffuse large B-cell lymphoma; EML4, echinoderm microtubule-associated protein-like 4; FN1, fibronectin 1; HIP1, huntingtin interacting protein 1; IMT, inflammatory myofibroblastic tumor; KIF5B, kinesin family member 5B; KLC1, kinesin light chain 1; MSN, moesin; MYH9, myosin heavy chain 9; N.D., not described; NPM1, nucleophosmin; NSCLC, non-small-cell lung cancer; PPFIBP1, PPFIA binding protein 1; PTPN3, protein tyrosine phosphatase, non-receptor type 3; RANBP2, RAN binding protein 2; RCC, renal cell carcinoma; RMC, renal medullary carcinoma; RNF213, ring finger protein 213; SEC31A, SEC31 Homolog A; SQSTM1, sequestosome 1; STRN, Striatin; TFG, TRK-fused gene; TPM3, tropomyosin 3; TPM4, tropomyosin 4; TPR, translocated promoter region, nuclear basket protein; TRAF1, TNF receptor associated factor 1; VCL, vinculin; WDCP, WD repeat and coiled coil containing.

**Table 2:** FDA Approved and new ALK inhibitors under development.

Inhibitor	Targeted Kinase/s	Activity against Mutant Forms	Clinical Evidence	Brain Penetrance	References
Crizotinib * (Xalkori–Pfizer)	ALKc-MET sROS1	EML4-ALK^L1198F^	Phase I Phase II Phase III s(Complete)	No	[9,10,101,102]
Ceritinib * s(Zykadia–Novartis)	ALK IGR-1R INSR STK22D	EML4-ALK^I1171T/N, L1196M, S1206C/Y, G1269A/S^	Phase I Phase II Phase III (NCT02393625)	Yes	[11,109,110,111]
Alectinib * (Alecensa–Roche)	ALK LTK GAK	EML4-ALK^L1152P/R, C1156Y/T, L1196M, F1174C/Y, S1206C/Y^DCTN1-ALK^G1269/S^	Phase I Phase II Phase III (NCT02075840)	Yes	[14,116,117,127,129,130]
Brigatinib * (AP26113-Ariad)	ALK ROS1	EML4-ALK^I1151Tins, C1156Y/T, L1196M, L1152P/R, F1174C/L/V, G1269A/S^^1^ EML4-ALK^G1202R^	Phase I Phase II Phase III (NCT02094573)	Yes	[16,121,123,124,125,131,132,133]
PF-06463922 (Lorlatinib-Pfizer)	ALK ROS1	ROS1^G2032R^ ROS1^L2026M^ EML4-ALK^L1196M, G1269A,S1206Y,C1156Y,F1174L,L1152R,1151Tins^	Phase I Phase II (NCT01970865) Phase III (NCT03052608)	Yes (NCT02927340)	[8,127,134,135,136]
RXDX-101 (Entrectinib-Ignyta)	ALK ROS1 TrkA TrkB TrkC	EML4-ALK^C1156Y, L1196M^	Phase I (ALKA-372-001 and STARTRK-1; NCT02097810)	Yes	[137,138]
ASP3026 (Astellas Pharma)	ALK ACK ROS1	EML4-ALK^L1196M^ NPM-ALK^I231N^ NPM-ALK^L256Q^	Phase I (NCT01284192)	N.D.	[139,140,141]
X-376 and X-396 (Xcovery)	ALK MET	EML4-ALK^L1196M, C1156Y^	Phase I/II (X-396) (NCT01625234)	Yes	[142,143]
CEP-28122 (Teva)	ALK FAK	N.D.	Phase I (NCT01922752)	N.D.	[144]
TSR-011 (Tesaro)	ALK TrkA TrkB TrkC	N.D.	Phase I/IIa (NCT02048488)	N.D.	[145]

Abbreviations (alphabetic order): FAK, focal adhesion kinase; MET, proto-oncogene, receptor tyrosine kinase; N.D., not described; ROS1, ROS proto-oncogene 1, receptor tyrosine kinase; TrkA, tyrosine kinase receptor A; TrkB, tyrosine kinase receptor B; TrkC, tyrosine kinase receptor C. *; FDA approved. ^1^ Brigatinib was reported to have activity against the G1202R mutation [124,125,146], however, G1202R mutation has also been detected in biopsy specimens from ALK-positive NSCLC patients who relapsed on brigatinib [129].

**Table 3:** Mutational profile of ALK that induce TKI resistance.

TKI	Sensitive Mutants	Resistant Mutants	Disease	Evidence (In Vitro/In Vivo/Clinical)	Reference
**Crizotinib**	L1198F	I1151Tins L1152R C1156Y I1171T/N F1174L L1196M L1196Q L1198P G1202R D1203N S1206Y G1269A	NSCLC NSCLC NSCLC NSCLC IMT NSCLC NSCLC EML4-ALK BaF3 cells NSCLC NSCLC NSCLC NSCLC, IMT	Clinical Clinical Clinical Clinical Clinical Clinical Clinical In vitro Clinical Clinical Clinical Clinical	[158] [156] [148] [160] [156] [148] [161] [162] [158] [161] [158] [159,163]
**Ceritinib**	G1269A, I1171T, S1206Y, L1196M	R1275Q L1152P/R D1203 G1202R F1174C/V L1198F C1156Y/T	Neuroblastoma NSCLC NSCLC NSCLC NSCLC NSCLC NSCLC	In vitro In vitro Clinical Clinical Clinical In vitro In vitro	[94] [107] [164] [107] [107] [165] [107]
**Alectinib**	G1269A, S1206Y, L1152R, F1174L, 1151Tins	I1171T V1180L G1202R	NSCLC NSCLC NSCLC	Clinical In vitro Clinical	[119] [155]
**Brigatinib**	G1269A, S1206Y, L1152R, F1174C, 1151Tins, I1171T, D1203N, E1210K, F1245C	F1174V+L1198F G1202R S1206C/F	ALCL NSCLC NSCLC	In vitro Clinical Clinical	[166] [167]
**Lorlatinib**	L1196M, G1202R, G1269A	L1198F	NSCLC	Clinical	[147]
